# Mutations in spliceosome genes in myelodysplastic neoplasms and their association to ring sideroblasts

**DOI:** 10.1038/s41375-022-01783-y

**Published:** 2022-12-03

**Authors:** Sandra Huber, Torsten Haferlach, Manja Meggendorfer, Stephan Hutter, Gregor Hoermann, Isolde Summerer, Irene Fuhrmann, Constance Baer, Wolfgang Kern, Claudia Haferlach

**Affiliations:** MLL Munich Leukemia Laboratory, Max-Lebsche-Platz 31, 81377 Munich, Germany

**Keywords:** Cancer genetics, Myelodysplastic syndrome

## To the Editor:

Two new proposals for the classification of myeloid malignancies have been presented: the 5th edition of the WHO Classification (WHO 2022 [1]) and the International Consensus Classification (ICC [2]). Here we address differences in entity defining criteria within myelodysplastic neoplasms (MDS), in particular in MDS with low blasts, and discuss accompanying hurdles.

MDS is a very heterogeneous disease representing clonal disorders of hematopoietic cells characterized by morphologic dysplasia, peripheral cytopenias, ineffective hematopoiesis and increased risk of leukemic transformation [3]. Somatic mutations in splicing pathway genes are detected in about half of MDS patients with *SF3B1* as the most commonly mutated one, typically found in MDS with ring sideroblasts (RS) [4, 5]. *SF3B1* mutations further define a distinct MDS subtype showing favorable prognosis and indolent disease course [6, 7]. Thus, MDS with low blasts and *SF3B1* mutation (MDS-*SF3B1*) is considered a separate MDS entity in both WHO 2022 [1] and ICC [2] with slightly deviating defining entity criteria (Suppl. Table S1). Both WHO 2022 and ICC classifications require the presence of an *SF3B1* mutation (WHO: VAF ≥ 5%, ICC: VAF ≥ 10%), a bone marrow (BM) blast count <5% and the absence of certain cytogenetic abnormalities and biallelic *TP53* inactivations. The ICC further requires the absence of *RUNX1* mutations. In contrast to ICC, in WHO 2022 the term “MDS with low blasts and ring sideroblasts” (MDS-LB-RS) is retained as an acceptable alternative to be used for cases with wild-type *SF3B1* and ≥15% ring sideroblasts allowing the inclusion of driver mutations in other splicing components.

Here, we address the differences between WHO and ICC regarding MDS-*SF3B1* and in particular if the WHO term “MDS with low blasts and ring sideroblasts” is meaningful as an alternative for *SF3B1* wild-type cases. Therefore, we analyzed this “alternative group” referred to as MDS-LB-RS in an MDS cohort with respect to incidence, presence of other splicing gene mutations and clinical outcome.

We selected 704 *de novo* MDS patients with sample material available to perform WGS sent to our laboratory between 09/2005 and 12/2019 (male/female: 407/297; median age: 73 [23–93]; 409 with <5% BM blasts). Diagnoses were made based on cytomorphology, cytogenetics and molecular genetics as previously published [8]. All samples were subjected to amplification-free WGS (median coverage >100x) as reported previously [9, 10]. The validation cohort comprised 1804 *de novo* MDS patients (male/female: 1160/644; median age: 76 [24–96]; 1015 with <5% BM blasts) whose samples were subjected to targeted panel sequencing during routine diagnostics between 07/2017 and 07/2022 as previously described [11]. Details on statistics see supplement. All patients had given written informed consent to the use of genetic and clinical data according to the Declaration of Helsinki and the study was approved by the internal review board.

**Fig. 1:**
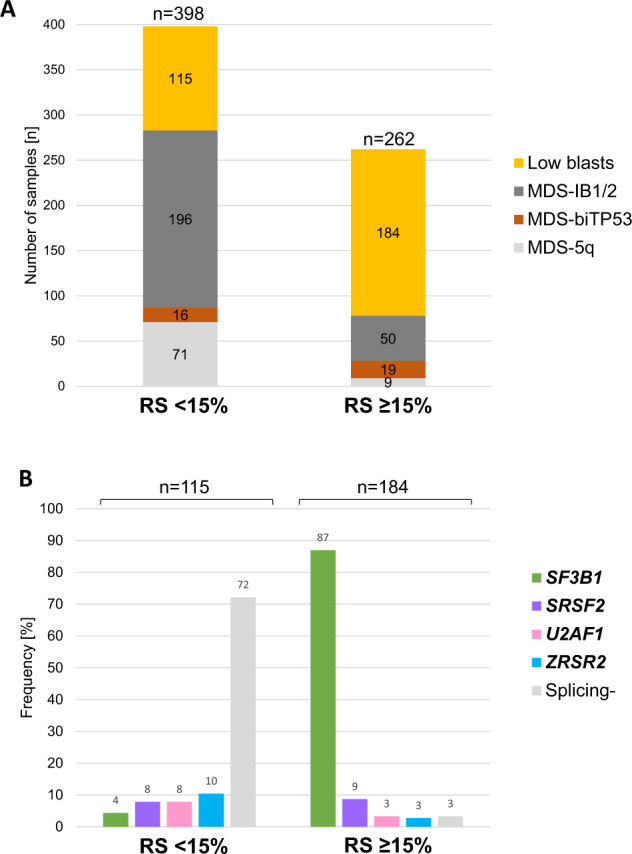
MDS cohort overview according. **A** Distribution of subsets within MDS patients according to WHO 2022 dependent on the presence of ring sideroblasts (RS). **B** Frequency of mutations in splicing genes *SF3B1, SRSF2, U2AF1* or *ZRSR2* within low blast MDS with RS < 15% or ≥15%. Splicing-: no mutation in splicing genes *SF3B1, SRSF2, U2AF1* or *ZRSR2*.

In 660/704 (94%) MDS cases data on the presence of RS were available and the basis for further analyses. Of these, 40% (262/660) showed RS ≥ 15% (Fig. 1A). 299/660 patients had low blasts (LB; BM blasts <5%) and also did not fulfill the criteria for the WHO 2022 entities MDS with low blasts and isolated 5q deletion (MDS-5q; *n* = 98) or MDS with biallelic *TP53* inactivation (MDS-bi*TP53*; *n* = 41). RS < 15% were detected in 115/299 (38%) while 184/299 (62%) patients showed RS ≥ 15%.

In LB cases with RS ≥ 15% splicing mutations were found in 178/184 (97%) (*SF3B1:* 87%*, SRSF2:* 9%*, U2AF1:* 3%*, ZRSR2:* 3%), while only 28% (32/115) of patients with LB and RS < 15% harbored mutations in at least one of four analyzed splicing genes (*SF3B1:* 4%*, SRSF2:* 8%*, U2AF1:* 8%*, ZRSR2:* 10%; Suppl. Fig. S1A; Fig. 1B). Next, we analyzed the association of the distinct splicing gene mutations (*SF3B1, SRSF2, U2AF1, ZRSR2*) with the presence of RS (<15% vs. ≥15%) and BM blasts (<5% vs. ≥5%). *SF3B1* mutations were significantly associated with BM blasts <5% (*p* < 0.001), while mutations in all other splicing genes showed association with BM blasts ≥5% (*SRSF2*: *p* < 0.001, *U2AF1*: *p* = 0.006, *ZRSR2*: *p* = 0.083; Supplementary Fig. S1B). These observations were mirrored in the large validation cohort (*SF3B1*: *p* < 0.001, *SRSF2*: *p* < 0.001, *U2AF1*: *p* = 0.003, *ZRSR2*: *p* = 0.037) where the trend in *ZRSR2* reached statistical significance (Suppl. Fig S1B). We found that *SF3B1* mutations were significantly associated with RS ≥ 15% (*p* < 0.001), while mutations in *SRSF2, U2AF1* and *ZRSR2* were not. For *SRSF2* (*p* = 0.056) and *ZRSR2* (*p* = 0.016) mutations we even observed a trend towards an association with RS < 15%. This was again confirmed in our validation cohort (*SF3B1*: *p* < 0.001, *SRSF2*: *p* < 0.001, *ZRSR2*: *p* < 0.001) where the trend in *SRSF2* mutations became statistically significant. *U2AF1* mutations were not found to be associated with RS in either cohort (*p* = 0.130 and *p* = 0.125, respectively; Supplementary Fig S1B). Together, these data clearly indicate that other splicing factor mutations cannot be used as a substitute for *SF3B1* mutations and are not useful for the classification of MDS-LB-RS in the absence of *SF3B1* mutations.

In this line, 87% (160/184) of cases with RS ≥ 15% showed *SF3B1* mutations (Fig. 1B) indicating a high chance for detecting an *SF3B1* mutation if RS ≥ 15%. In cases with RS < 15%, only 4% (5/115) harbored an *SF3B1* mutation while mutations in *SRSF2*, *U2AF1* and *ZRSR2* were more frequent in this group (*SRSF2* and *U2AF1*: each 9/115; 8%; *ZRSR2*: 12/115; 10%). Thus on the other hand, 13% (24/184) of cases with RS ≥ 15% were *SF3B1* wild-type including two cases harboring complex karyotypes (Supplementary Fig. S2). Thus, 22 cases qualify to be assigned to MDS-LB-RS according to WHO 2022. In this group 17/22 (77%) cases harbored mutations in other spliceosome genes (*U2AF1*: *n* = 5, *SRSF2*: *n* = 12; VAF ≥ 10% in all cases; Suppl. Figure S2). Notably, in both RS groups (<15% vs. ≥15%) splicing gene mutations were not mutually exclusive, 13 cases harbored two splicing mutations (for details see Suppl. Results). Complex karyotypes and *RUNX1* mutations were detected in 5 and 10 cases with mutated splicing genes, respectively (Supplementary Fig. S2).

**Fig. 2:**
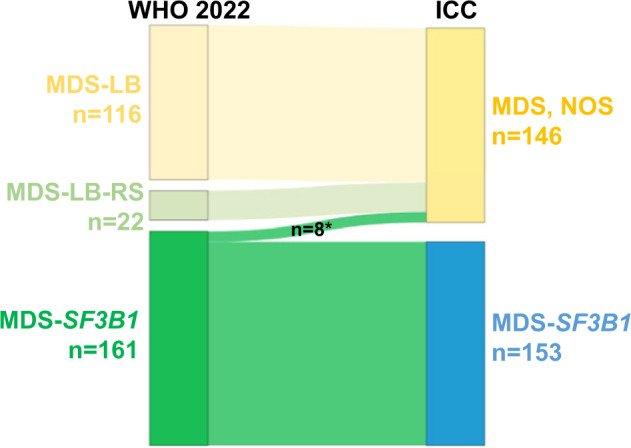
Changes in sample categorization within low blast MDS. Cases (*n* = 299) were categorized according to WHO 2022 (left) and ICC (right) classifications. VAF: variant allelic frequency; * *RUNX1*^mut^ (*n* = 7) and *SF3B1*^mut^ VAF < 10% (*n* = 1); mut mutation.

Differences in the defining entity criteria between WHO 2022 and ICC change the assignment into diagnostic categories of 30/299 MDS cases with low blasts (Fig. 2; Supplementary Fig. S3). The 22 cases classified as MDS-LB-RS according to WHO 2022 were classified as MDS, NOS based on ICC criteria. In addition, 8 cases assigned to the MDS-*SF3B1* entity according to WHO belong to MDS, NOS according to ICC due to presence of *RUNX1* mutations (*n* = 7) or *SF3B1* VAF < 10% (*n* = 1). To evaluate whether or not MDS-LB-RS according to WHO 2022 is associated with a comparable favorable outcome as MDS-*SF3B1* we performed survival analyses. Overall survival (OS) was significantly shorter in MDS-LB-RS (*n* = 22) compared to MDS-*SF3B1* (*n* = 161; median: 5.3 vs. 7.9 years; *p* = 0.032; Supplementary Fig. S4A), mainly mediated by MDS-LB-RS cases harboring splicing mutations (*p* = 0.046; Supplementary Fig. S4B, C). In addition, MDS-LB-RS showed comparable outcome to other MDS-LB (*n* = 116) not fulfilling the criteria for MDS-*SF3B1* or MDS-LB-RS (median: 5.3 vs. 6.2 years; *p* = 0.373; Supplementary Fig. S4D) and thus grouped together showed shorter OS compared to the MDS-*SF3B1* entity (median: 5.8 vs. 7.9 years; *p* = 0.038; Supplementary Fig. S4E).

Of note, within the WHO MDS-*SF3B1* entity (*n* = 161) *RUNX1* mutated cases (*n* = 7) were associated with shorter OS compared to *RUNX1* wild-type cases (median: 2.1 vs. 8.3 years; *p* < 0.001; Supplementary Fig. S4F; for details on further co-mutations see Supplementary Results). Excluding *RUNX1* mutated cases from WHO MDS-*SF3B1* led to a more significant separation regarding OS between MDS-*SF3B1* cases and non-MDS-*SF3B1* cases with LB (*p* = 0.003 vs. *p* = 0.038; Supplementary Fig. S4G/E). Based on ICC, excluding *RUNX1* mutations and *SF3B1* VAFs <10% from the MDS-*SF3B1* entity, a similar significant separation regarding OS was achieved between ICC MDS-*SF3B1* cases and non-MDS-*SF3B1* cases represented by MDS, NOS (*p* = 0.005; Supplementary Fig. S4H).

Following this, with regard to prognosis excluding *RUNX1* mutations from the prognostically favorable MDS-*SF3B1* entity is rational, concordant with the proposal of the IWG-PM [7]. In this line, several studies demonstrated the negative prognostic value of *RUNX1* co-mutations in *SF3B1* mutated MDS [6, 12–14], thereby highlighting the role as potential driver gene associated with worse OS and a higher leukemic transformation rate within *SF3B1* mutated patients [15]. In addition, we previously showed that in *SF3B1* mutated MDS del(5q) and/or *RUNX1* mutations have a negative impact on outcome while a BM blast threshold of <5%, which is used by ICC and WHO 2022, has no independent impact on OS [15].

In conclusion, we again confirmed the association of *SF3B1* mutations with low BM blasts and increased RS. We also showed that mutations in other splicing genes (*SRSF2* and *ZRSR2*) were significantly associated with high BM blasts and low RS. Thus, we suggest the alternative term “MDS with low blasts and ring sideroblasts” (MDS-LB-RS) as a second best classification only for cases when *SF3B1* mutation analysis is not available as in this setting RS ≥ 15% represent a good but not perfect surrogate for *SF3B1* mutations. Conversely, MDS-LB-RS with wild-type *SF3B1* are suggested to be classified as MDS-LB and separated from MDS-*SF3B1*, as biology seems to be different as indicated by a less favorable prognosis. We further demonstrated the negative prognostic impact of *RUNX1* mutations in *SF3B1* mutated patients and therefore suggest excluding these cases from the MDS-*SF3B1* entity.

## Supplementary information

Supplemental Material

## Data Availability

The datasets generated during and/or analyzed during the current study are available from the corresponding author on reasonable request.
