# Supplementary material for: Mutations in spliceosome genes in myelodysplastic neoplasms and their association to ring sideroblasts
Source: Leukemia. 2022 Dec 3;37(2):500–2. doi: 10.1038/s41375-022-01783-y (PMC9898028; doi:10.1038/s41375-022-01783-y)
Supplement: Supplementary file 1 — Supplemental Material [file 41375_2022_1783_MOESM1_ESM.pdf]

Supplemental Material

Supplementary Text

Methods

Statistical analysis

Statistical analyses were performed using SPSS version 19.0 (IBM Corporation, Armonk, NY). Analyses for overall survival (OS) was performed according to Kaplan-Meier and compared using two-sided log rank tests. Dichotomous variables were compared using chi-square test. All results were considered significant at $p<0.05$.

Results

Cases with multiple splicing mutations

Notably, 10/13 cases with multiple splicing mutations showed RS ≥15%, all harboring *SF3B1* mutations (Suppl. Figure S2B; Suppl. Table S2; *SF3B1+ZRSR2*: n=6; *SF3B1+SRSF2*: n=3; *SF3B1+U2AF1*: n=1). We did not find copy loss or CN-LOH at any splicing gene loci in any of the 10 cases. Based on the VAFs, it was not clear in 6 cases whether both mutations appeared in the same clone or in different, independent clones. However, in 4 cases the VAFs of both splicing mutations indicated their occurrence in the same clone. Of those 4 cases, the *SF3B1* mutation seemed to be in the dominating clone in two cases, while in one case *ZRSR2* was the dominating mutation. In another case *SF3B1* and *SRSF2* showed similar VAFs.

Additional co-mutations in SF3B1 mutated patients

We further analyzed the *SF3B1* mutated cases (without complex karyotypes and *SF3B1* VAF ≥5% as suggested by WHO 2022; n=161) for the presence of mutations in *BCOR, BCORL1, NRAS, STAG2* and *SRSF2,* as they were previously described as unfavorable risk factors in *SF3B1* mutated patients [1]. In total, we found 8 cases with

corresponding mutations (*BCOR*: n=3, *BCORL1*: n=0, *NRAS*: n=0, *STAG2*: n=2, *SRSF2*: n=3). *SF3B1* mutated patients with additional *BCOR, STAG2* or *SRSF2* mutations showed a trend towards shorter OS (median OS: 5.6 vs. 8.4 years; p=0.205; Suppl. Figure S4J), however not reaching statistical significance presumably due to the small samples size.

Supplementary Tables

Table S1. Classification and entity criteria of MDS with *SF3B1* mutations

	**5th edition of WHO [A]**	**ICC [B]**
**Criteria**	MDS with low blasts and *SF3B1* mutation*	MDS with mutated *SF3B1*
Cytopenia	≥1	≥1
Dysplasia	≥1	**Not required**
Blasts	<5% BM; <2% PB	<5% BM; <2% PB
Cytogenetics	Absence of 5q deletion, monosomy 7, or complex karyotype **	Absence of isolated del(5q), -7/del(7q), abn3q26.2, or complex
Mutations	*SF3B1* (**VAF ≥5%**) without biallelic *TP53* **	*SF3B1* (**VAF ≥10%**) without ***RUNX1*** or multi-hit *TP53*
Ring sideroblasts (RS)	Not required	Not required

* Possible substitution of *SF3B1* mutation by detecting ≥15% RS. Acceptable related terminology: MDS with low blasts and ring sideroblasts.

** Excluding AML-defining genetic abnormalities

[A] Khoury *et al.,* Leukemia, 2022; 36(7):1703-19; Variant allelic frequency (VAF) criteria retrieved from *https://tumourclassification.iarc.who.int/chaptercontent/63/23.* Accessed 06 Sep 2022. [B] Arber *et al*., Blood, 2022; 140(11):1200-28.

Table S2. MDS cases with low blasts and RS ≥15% with multiple splicing mutations

Sample	*SF3B1* VAF	*ZRSR2* VAF	*SRSF2* VAF	*U2AF1* VAF
**#1**	47%	31%		
**#2**	47%	5%		
**#3**	9%	55%		
**#4**	33%	4%		
**#5**	32%	7%		
**#6**	15%	19%		
**#7**	44%		41%	
**#8**	31%		4%	
**#9**	42%		4%	
**#10**	29%			13%

VAF: Variant allelic frequency

Supplementary Figures

A

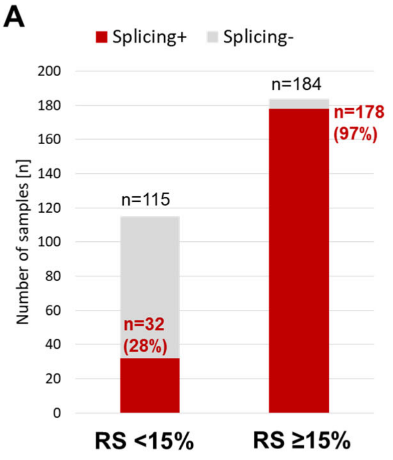

B

		BM blasts <5%	BM blasts ≥5%	*P*	RS <15%	RS ≥15%	*P*
SF3B1	n=660	185/391 (47%)	35/269 (13%)	***p*<0.001**	24/398 (6%)	196/262 (75%)	***p*<0.001**
	n=1804	437/1015 (43%)	75/789 (10%)	***p*<0.001**	85/1127 (8%)	427/677 (63%)	***p*<0.001**
SRSF2	n=660	26/391 (7%)	71/269 (26%)	***p*<0.001**	67/398 (17%)	30/262 (11%)	*p*=0.056
	n=1804	153/1015 (15%)	203/789 (25%)	***p*<0.001**	276/1127 (24%)	80/677 (12%)	***p*<0.001**
U2AF1	n=660	15/391 (4%)	24/269 (9%)	***p*=0.006**	28/398 (7%)	11/262 (4%)	*p*=0.130
	n=1804	85/1015 (8%)	100/789 (13%)	***p*=0.003**	106/1127 (9%)	79/677 (12%)	*p*=0.125
ZRSR2	n=660	19/391 (5%)	22/269 (8%)	*p*=0.083	32/398 (8%)	9/262 (3%)	***p*=0.016**
	n=1804	55/1015 (5%)	62/789 (8%)	***p*=0.037**	103/1127 (9%)	14/677 (2%)	***p*<0.001**

Supplementary Figure S1: Frequency and association of splicing gene mutations in MDS. (A) Distribution and frequency of splicing genes *SF3B1, SRSF2, U2AF1* and *ZRSR2* (red: mutated = splicing+; grey: wild-type = splicing-) within low blast MDS (n=299) grouped into harboring ring sideroblasts (RS) <15% or ≥15%. **(B)** Association of *SF3B1, SRSF2, U2AF1* and *ZRSR2* mutations with BM blasts (<5% vs. ≥5%) and RS (<15% vs. ≥15%) within MDS cases with available RS data (n=660) and an independent validation cohort (n=1804); *p*-values in bold: statistical significance (*p*≤0.05).

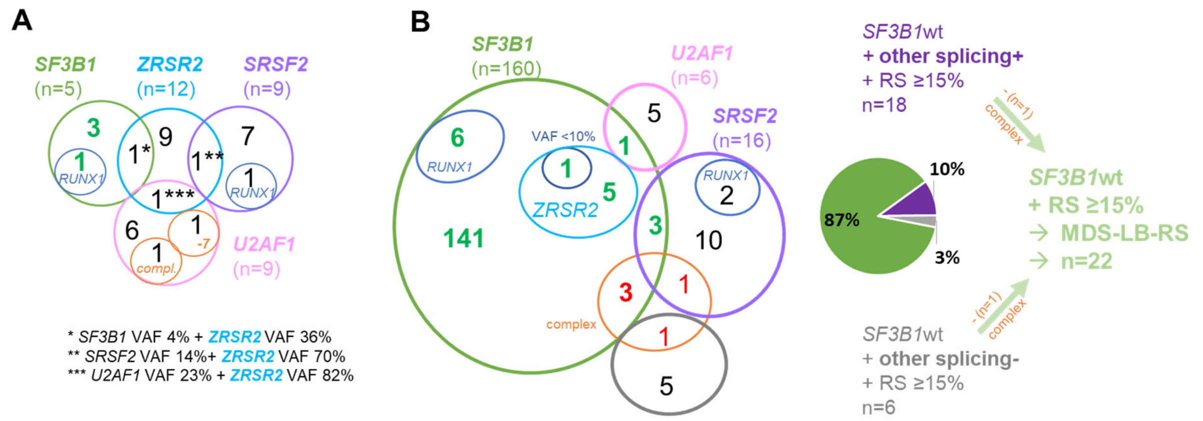

Supplementary Figure S2: Cases with splicing gene mutations in low blast MDS and their additional genetic abnormalities. (A) Number of cases with splicing gene mutations in low blast MDS with RS <15% (n=115). compl.: complex karyotype; -7: monosomy 7; *RUNX1*: additional *RUNX1* mutation; VAF: variant allelic frequency; **(B)** Number and frequency of cases with splicing gene mutations in low blast MDS with RS ≥15% (n=184) and the categorization of samples into MDS-LB-RS according to WHO 2022. complex: complex karyotype; *RUNX1*: additional *RUNX1* mutation; wt: wild-type; other splicing+/-: with (+) / without (-) mutation in splicing genes *SRSF2, U2AF1* or *ZRSR2*. VAF: variant allelic frequency.

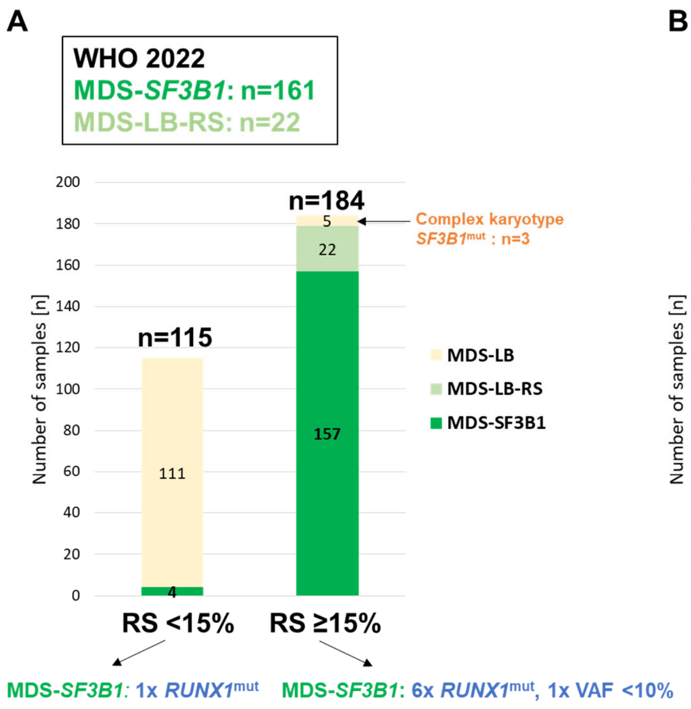

Supplementary Figure S3: Classification and distribution of subsets within low blast MDS (n = 299). (A) Cases were categorized according to WHO 2022 and grouped into ring sideroblasts (RS) <15% or ≥15%. VAF: variant allelic frequency; mut: mutation. **(B)** Cases were categorized according to ICC and grouped into ring sideroblasts (RS) <15% or ≥15%.

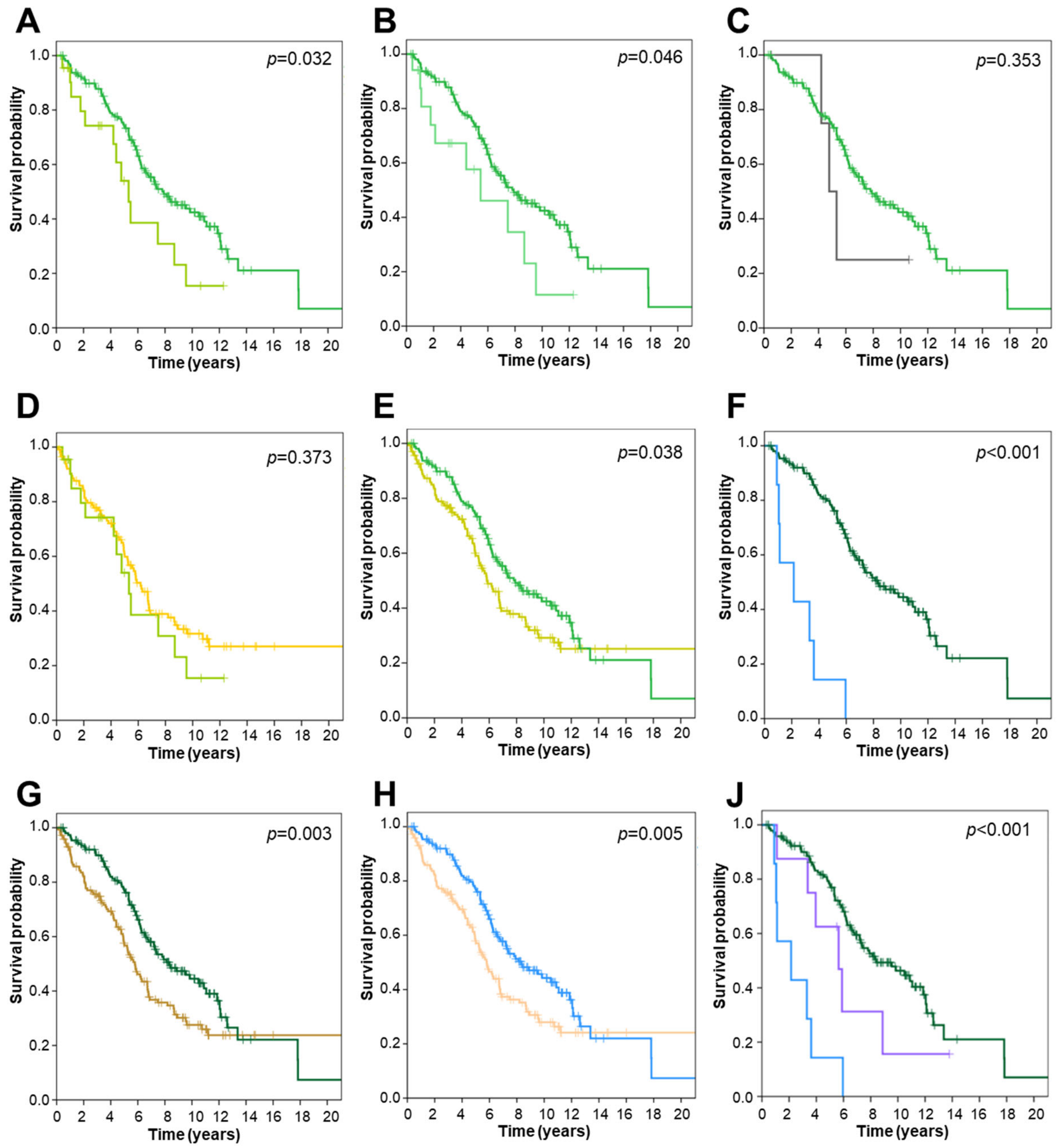

Supplementary Figure S4: Kaplan-Meier plots of different subsets within low blast MDS patients. (A) Overall survival (OS) of MDS-*SF3B1* (n=161; green) compared to MDS-LB-RS (n=22; neon green) according to WHO 2022. **(B)** OS of MDS-*SF3B1* (n=161; green) compared to MDS-LB-RS harbouring splicing mutations (n=17; light green) according to WHO 2022. **(C)** OS of MDS-*SF3B1* (n=161; green) compared to MDS-LB-RS without splicing mutations (n=5; grey) according to WHO 2022. **(D)** OS of MDS-LB (n=116; yellow) compared to MDS-LB-RS (n=22; light green) according to WHO 2022. **(E)** OS of MDS-*SF3B1* (n=161; green) compared to non-MDS-*SF3B1* represented by MDS-LB and MDS-LB-RS (n=138; light brown) according

to WHO 2022. **(F)** OS of WHO MDS-*SF3B1* without additional *RUNX1* mutations (n=154; dark green) compared to with additional *RUNX1* (n=7; blue). **(G)** OS of WHO MDS-*SF3B1* without additional *RUNX1* mutations (n=154; dark green) compared to remaining low blast MDS (n=145; brown). **(H)** OS of MDS-*SF3B1* (n=153; blue) compared to MDS, NOS (n=146; rose) according to ICC. **(J)** OS of WHO MDS-*SF3B1* with additional *RUNX1* (n=7; blue) or with additional *BCOR, STAG2* or *SRSF2* mutations (n=8; purple) compared to cases without corresponding additional mutations (n=146; dark green).

References

1. Bernard E, Tuechler H, Greenberg Peter L, Hasserjian Robert P, Arango Ossa Juan E, Nannya Y, et al. Molecular International Prognostic Scoring System for Myelodysplastic Syndromes. NEJM Evidence. 2022;1(7):EVIDoa2200008.
